# A systematic review of ethical issues in vaccine studies involving pregnant women

**DOI:** 10.1080/21645515.2016.1186312

**Published:** 2016-05-31

**Authors:** Jennifer A. Beeler, Philipp Lambach, T. Roice Fulton, Divya Narayanan, Justin R. Ortiz, Saad B. Omer

**Affiliations:** a Hubert Department of Global Health, Rollins School of Public Health, Emory University, Atlanta, GA, USA; b Initiative for Vaccine Research, World Health Organization, Geneva, Switzerland; c Department of Epidemiology, Rollins School of Public Health, Emory University, Atlanta, GA, USA; d Department of Pediatrics, Emory University School of Medicine, Atlanta, GA, USA; e Emory Vaccine Center, Emory University, Atlanta, GA, USA

**Keywords:** ethics, maternal immunization, vaccinology

## Abstract

**Background**: Immunization during pregnancy can provide protection for mother and child. However, there have been only a limited number of studies documenting the efficacy and safety of this strategy. **Aims**: To determine the extent and nature of subject matter related to ethics in maternal immunization by systematically documenting the spectrum of ethical issues in vaccine studies involving pregnant women. **Method**: We conducted a systematic literature review of published works pertaining to vaccine and therapeutic studies involving pregnant women through searches of PubMed, EMBASE, Web of Science, the Cochrane Database, and ClinicalTrials.gov. We selected literature meeting the inclusion criteria published between 1988 and June 2014. We systematically abstracted subject matter pertaining to ethical issues in immunization studies during pregnancy. Immunization-specific ethical issues were matched and grouped into major categories and subcategories. **Results**: Seventy-seven published articles met the inclusion criteria. Published articles reported findings on data that had been collected in 26 countries, the majority of which were classified as high-income or upper-middle-income nations according to World Bank criteria. Review of these publications produced 60 immunization-specific ethical issues, grouped into six major categories. Notably, many studies demonstrated limited acknowledgment of key ethical issues including the rights and welfare of participants. Additionally, there was no discussion pertaining to the ethics of program implementation, including integration of maternal immunization programs into existing routine immunization programs. **Conclusion**: This review of ethical issues in immunization studies of pregnant women can be used to help inform future vaccine trials in this important population. Consistent documentation of these ethical issues by investigators will facilitate a broader and more nuanced discussion of ethics in immunization of pregnant women – offering new and valuable insights for programs developed to prevent disease in newborn children in low- and middle-income countries.

## Introduction

Immunization is one of the most powerful and cost-effective interventions against infectious diseases.^1^ Immunization of pregnant women (“maternal immunization”) has the potential to protect mothers, fetuses, and infants from infectious diseases, as well as prevent complications due to maternal infection during prenatal development.^2,3^

A growing number of low- and middle-income countries are considering adapting national policies to include seasonal influenza vaccination in their schedules to protect pregnant women.^4,5^ In 2012, the WHO Strategic Advisory Group of Experts on Immunization (SAGE) recommended the prioritization of influenza vaccination of pregnant women at any stage of pregnancy for countries considering initiating or expanding influenza vaccine programs.^6^ Similarly, SAGE recommends immunization of pregnant women, in certain circumstances, against *Clostridium tetani* (tetanus), *Bordetella pertussis* (pertussis), and *Neisseria meningitidis*. Recently, the Global Advisory Committee on Vaccine Safety reviewed the use of numerous vaccines (including but not limited to inactivated influenza, tetanus toxoid, and meningococcal polysaccharide and conjugated vaccines) during pregnancy and found no adverse pregnancy outcomes identified from immunization.^7^ However, theoretical risks associated with live attenuated vaccines remain.

While the body of literature regarding the safety and effectiveness of maternal immunization is growing,^7,8^ there are relatively few clinical trials or prospective studies that include pregnant women. Pregnant women and infants are generally classified as vulnerable subjects, and are often excluded from clinical trials. Investigators are often reluctant to include pregnant women, as there are additional health concerns, and a need to avoid unnecessary risk to the fetus.^9,10^ Informing researchers of the ethics of including vulnerable subjects in controlled trials may increase willingness to include pregnant women and infants in safety studies.

There is a considerable knowledge gap regarding ethical issues in vaccine studies – particularly those where vaccines are administered during pregnancy. Several crosscutting themes concerning ethics in maternal immunization have emerged. The more critical of these themes are the need for public justification and transparency, proper framing of information, and the importance of ensuring quality routine care delivery with the introduction of new interventions into the antenatal care platform.^11,12^ The primary aim of this review is to determine the scope of ethical subject matter in articles of vaccine trials among pregnant women.

## Results

Seventy-seven publications published between 1988 and 2014 met the criteria for inclusion. More than half of the selected studies (39) focused on influenza vaccines, followed by yellow fever (5), Tdap (5), and rubella vaccines (4) (Table 1). Of the 39 influenza studies that met inclusion criteria, the majority (22) focused on H1N1, a smaller number (12) focused on seasonal (non-pandemic) influenza, while the remaining studies (5) considered both H1N1 and seasonal influenza vaccines. The large number of studies specific to the H1N1 vaccine is likely in response to the 2009 pandemic. No systematic differences in ethical discussion between seasonal and pandemic flu vaccines were identified. Additional studies investigated the use of tetanus toxoid, as well as vaccines protecting against cholera, cytomegalovirus, group B streptococcus, hepatitis, herpes simplex, human immunodeficiency virus (HIV), human papillomavirus (HPV), meningococcal disease, pneumococcal disease, polio, rabies, respiratory syncytial virus, and varicella. Five studies investigated multiple vaccines. The majority of studies were conducted in North America (36) or Europe (15), with the remaining studies being conducted in Asia (8), South America (7), and Africa (2). Nine studies were conducted in multiple countries (Table 1). Additionally, the majority of studies (excluding systematic reviews and meta-analyses) were conducted in high- or middle-income countries, with 36 having been conducted in the United States or Puerto Rico (Table 2). Notably, only two studies were conducted in low-income countries. Publications were either written or available in English language. Table S1 details all studies that met the inclusion criteria.

**Table 1.** Summary characteristics of selected studies (n = 77).

Selected publications by type	
Study type	Number of papers
Retrospective Cohort	27
Prospective Cohort	24
Randomized Controlled Trial (RCT)	13
Review	8
Cross-Sectional	2
Case-Control	2
Before/After	1
Selected publications by location	
Continent	Number of papers
North America	36
Europe	15
Asia	8
South America	7
Africa	2
Australia	0
Multiple	9
Selected publications by vaccine	
Vaccine	Number of papers
Influenza	39
Yellow Fever	5
Tdap	5
Rubella	4
Varicella	2
Group B streptococcus	2
Hepatitis	2
HIV	2
Pneumococcal	2
HPV	1
Cholera	1
Cytomegalovirus	1
Herpes simplex	1
Meningococcal	1
Polio	1
Rabies	1
Respiratory syncytial virus	1
TT	1
Multiple	5

**Table 2.** World Bank income classification of study countries (excluding systematic reviews).

High-Income (≥$12,746)	Canada (3)
	Chile (1)
	Denmark (2)
	Finland (1)
	France (5)
	Germany (1)
	Italy (1)
	Japan (1)
	Netherlands (2)
	Sweden (2)
	Taiwan (2)
	United Kingdom (2)
	United States (including Puerto Rico) (36)
	Uruguay (1)
Upper-Middle-Income ($4,126 to $12,745)	Argentina (3)Brazil (6)China (1)
	Ecuador (1)
	Peru (1)
	Thailand (1)
	Turkey (1)
	Venezuela (1)
Lower-Middle-Income ($1,046 to $4,125)	Papua New Guinea (1)Philippines (1)
Low Income (≤$1,045)	Bangladesh (1)
	Tanzania (1)

Publications meeting the inclusion criteria included a spectrum of 60 unique ethical issues that we grouped into six major categories: ethical conduct and institutional review board approval; disclosure and informed consent procedures; decision-making factors; cultural considerations; inadvertent immunization during pregnancy; and study design and enrollment (Appendix). Four of the six major categories were explicitly related to study design and implementation. For each major category, identified ethical issues were classified as first- or second-order categories. Broader ethical issues were classified as first-order categories, while specific ethical issues were classified as second-order categories. Analysis identified 54 unique second-order categories, which were grouped under 23 first-order categories (Appendix). For example, the major category ‘cultural considerations’ consisted of four second-order ethical issues grouped into three first-order categories. ‘Role of local leaders, community members, and relatives’ was an example of a first-order category, under which ‘community informed consent from local leaders’ was classified as a second-order ethical issue. Additionally, three second-order ethical issues were grouped under multiple first-order categories. For example, ‘community informed consent from local leaders’ was classified under two first-order ethical issues, as it was relevant to both ‘Disclosure and Informed Consent Procedures’ and ‘Cultural Considerations.’

## Discussion

In a 2015 meeting, SAGE highlighted the importance of the maternal immunization platform, and emphasized the need to strengthen vaccine delivery during pregnancy, particularly for high-risk groups worldwide.^13^ Little published research has focused on describing ethical issues in vaccine studies involving pregnant women. Much of the discussion related to ethics in maternal immunization studies has focused on pregnant women, fetuses, and neonates being classified as vulnerable subjects in human-subject research.^10^ However, there is a need and a growing interest in expanding this discussion in order to inform future research, regulations, and implementation of immunization programs.

## Spectrum of ethical issues in maternal immunization

In this review, we documented the spectrum of ethical issues in vaccine trials involving pregnant women, as described in the scientific literature. We found the spectrum to be rather narrow, neglecting key ethical issues. Additionally, we found the depth of relevant discussion to be somewhat cursory.

Sixty unique ethical issues were identified in our analysis in the 77 publications included in our review. A substantial proportion of the ethical issues identified in this review were specifically related to study design and implementation, review board processes, information and risk disclosure, and informed consent procedures. However, discussion involving information disclosure and informed consent procedures varied significantly. The majority of studies explicitly stated that written informed consent had been obtained, or that informed consent had been waived when deemed appropriate by an ethics review committee. Only one publication provided detailed information regarding informed consent protocols, including the multitude of ways in which informed consent was obtained, and how it was documented and maintained throughout the duration of the study.^14^ Collectively, discussion of informed consent procedures in publications included in this review demonstrates the variation in these processes.

The theoretical risks of vaccination for the fetus, and the lack of clear information regarding vaccine safety for some vaccines, are often cited as barriers to expanding maternal immunization programs.^2,15^ However, none of the publications meeting the inclusion criteria explicitly discussed how these risks and other ethical challenges may have impacted study enrollment. Several studies cited concerns of vaccine safety among pregnant women, providers, and study investigators.^2,16-29^ Yet, no publications included in this review detailed how perceived safety concerns among participants were addressed during enrollment, and only one study included in this review discussed how safety concerns affected study participation. In a prospective cohort of pregnant women exposed to rabies, study investigators reported that several participants withdrew from the study, or refused post-exposure prophylaxis due to perceived safety concerns.^20^ However, the need for further health education to eliminate perceived safety concerns was expressed.^20^

Also notable was the limited discussion of cultural considerations. Only four publications produced ethical issues classified under the cultural considerations major category.^14,20,27,30^ Two second-order ethical issues related to informed consent and decision-making processes were documented: one related to information disclosure, and one related to study enrollment. Yet, it is unlikely that these limited discussions represent the true breadth of ethical issues for this major category, as consent procedures and factors in decision-making will likely be unique to each study population and environment.

The need for public justification and transparency, framing of information, and integration into existing maternal and child health systems have been identified as three of the most prominent ethical considerations in maternal immunization.^11^ Additionally, considerations must include whether or not maternal immunization is a true priority, as well as risks and benefits of immunization for mother and child.^11^ Discussion of these key issues was largely absent in our included studies, leaving an element critical to the ethical implementation of maternal vaccine programs unaddressed.

There are numerous additional issues, which are not discussed in included studies, but are nevertheless critical. For example, the ethics of off-label vaccine use and the exclusion of pregnant women from trials are two issues that were not included in discussion of ethical issues in articles meeting the inclusion criteria for this review. Additionally, there was no discussion of whether or not it is ethical to develop and implement maternal immunization programs when few countries have maternal AEFI reporting systems. Discussion was also lacking with regard to maternal immunization in low- and middle-income countries (LMICs), issues of cost and access, and informed consent protocols, as well as how maternal immunization programs would be incorporated into existing immunization programs. Furthermore, the papers included in this review failed to discuss how a vertical maternal immunization system may disrupt routine immunization practices or antenatal care in LMICs.

Detailed discussion of how maternal immunization programs would be integrated into existing maternal, newborn, and child health services is imperative in order to prevent disruption of existing services.^31^ Additionally, there must be a cogent justification for the introduction of new vaccines in countries where current national vaccination coverage for Expanded Program on Immunization (EPI) vaccines is low.^31^ While the specific vaccine articles included in this review may not discuss these issues, they are crucial to the advancement of the maternal immunization agenda set forth by SAGE. It is notable, however, that there is very limited implementation research in maternal immunization, particularly in LMICs. The lack of discussion of these pertinent issues may indicate a need for greater involvement of ethicists in immunization.

## Limitations

Our study has several limitations. First, this literature review was restricted to English language publications meeting the inclusion criteria, published prior to June 2014. Ethical issues mentioned in studies published prior to 1988 were also excluded from this review. Therefore, ethical issues identified in this review may not represent the spectrum of ethical issues in vaccine trials involving pregnant women in studies conducted outside of this date range, or published in languages other than English. Second, our analysis was limited to ethical issues discussed in each publication, and did not include information that may have been omitted. Additional research, review of supplementary materials, and correspondence with study investigators could produce new categories of ethical issues that were not included in this report. Third, this review was limited to publications that met the inclusion criteria from a previous systematic review of AEFIs in vaccine studies involving pregnant women.^32^ Publications meeting the inclusion criteria consisted largely of RCTs, cohort studies, and literature reviews, and do not include any editorial or commentary pieces published in peer-reviewed journals or other sources (Table S1). As a result, the framework developed in this review does not necessarily represent the full scope of ethical issues discussed in all publications pertaining to maternal immunization. Lastly, data extraction and categorization were completed by a sole reviewer. While a concerted effort was made to ensure accuracy and completeness, it is possible that ethical issues may have been omitted, or categorized incorrectly.

## Future considerations

The data and findings from vaccine trials are necessary to advance our understanding of maternal immunization. However, they alone are insufficient to fully inform researchers and public health officials of the ethics of maternal immunization studies and programs. In documenting the spectrum of ethical issues in maternal immunization, we highlighted the need for greater and more in-depth discussion of ethics in vaccine studies involving pregnant women, as well as the need for additional clinical research specific to maternal immunization. Future reviews should consider conducting a scoping exercise using established frameworks and protocols, as scoping studies look at a wide range of evidence to convey breadth and depth of a particular field.^33^ Doing so would allow researchers to fully document the breadth of ethical issues discussed in all publications pertaining to maternal immunization, not just those identified in select randomized controlled trials, cohort studies, and literature reviews. New research should aim to refine and expand upon the framework developed in this review by highlighting additional ethical issues and omissions in the greater body of literature, in order to inform future immunization studies and programs involving pregnant women and newborns.

## Materials and methods

### Data sources and study selection criteria

Published studies related to immunization of pregnant women were identified in systematic searches of PubMed, EMBASE, Web of Science, the Cochrane Database, and ClinicalTrials.gov. Selected published works include randomized controlled vaccine trials, observational studies, and review articles, identified in a previous review.^32^ A detailed search strategy and study inclusion criteria for this review was derived from previous work, including a systematic review of vaccine safety data reporting ^34^ and a systematic review documenting the extent and variability in adverse events following immunization (AEFIs) in maternal and neonatal clinical trials.^32,34^ Study selection criteria are presented in Table 3.

**Table 3.** Criteria for study selection.

Participants	Pregnant women. Studies where pregnant women were inadvertently immunized were not excluded.
Intervention	Administration of one or more vaccines currently recommended for use during pregnancy, under consideration for recommendation, or previously recommended but later withdrawn.
Comparison Group	All relevant comparisons including, but not limited to, placebo/no-placebo, alternate vaccine formula, or alternate vaccine schedule.
Outcomes	Study outcomes included, but were not limited to drug efficacy, effectiveness, or safety.

### Defining immunization-specific ethical issues

In defining immunization-specific ethical issues (called “ethical issues” subsequently) we referred to established ethical and medical frameworks previously described in a review of ethical issues in dementia care.^35^ We referred to the theory of principlism, drawing from the ethical principles of beneficence, non-maleficence, respect for autonomy, and justice.^35^ We defined ethical issues as any subject matter pertaining to study-related information and disclosure, decision-making and consent, understanding of risks and benefits for mother and fetus, and a mother's understanding of the ability to give consent for herself and the fetus.

### Data extraction and categorization

Data extraction and categorization methods were adapted from a previously conducted study, which employed qualitative text and normative analysis to categorize the spectrum of ethical issues in clinical dementia care.^35^ The primary aim of this review was to develop a qualitative framework of broad and narrow categories representing the scope of ethical issues in vaccine studies involving pregnant women, rather than to document the frequency of ethical issues. In doing so, this review documents the breadth of ethical discussion in vaccine studies. One reviewer (JAB) reviewed all publications meeting the inclusion criteria. As there was limited room for subjective interpretation during the review process, only one reviewer was used, as agreed prospectively. Each article was reviewed twice to ensure accuracy and completeness in the process. We systematically identified and compared text that referenced ethical issues across all published articles. Similar ethical issues were grouped, and nested categories were constructed to best organize identified ethical issues. Categories of ethical issues were chosen based on the results of the issues identified through the review. All ethical issues were categorized by one reviewer (JAB) in order to maintain consistent categorization.

## Supplementary Material

Supplemental_Material.docx

## Appendix

The spectrum of immunization-specific ethical issues in vaccine studies involving pregnant women

1. Ethical Conduct and Institutional Review Board Approval
  Appropriate ethics approval:
  1. Institutional Review Board (IRB) approval.^14,18-20,22,24-28,36-56^
  2. Engagement with local and national ethics committees and participating study centers.^21,24,25,40-43,45,46,49,52,55-64^
  3. Secondary ethics approval.^61^
  Exemption from review:
  1. Does not meet definition of human research (VAERS, registry data, etc.).^54,65-71^
  2. Data anonymized.^72^
  3. Data extracted from published reports.^73^
  Conducted in accordance with standard ethical principles and national regulations
  1. National laws and federal regulations (ex. French law, US federal regulations governing protection of human subjects in research).^39,74^
  2. Declaration of Helsinki.^14,55,74^
  3. Principles of Good Clinical Practice.^55,61^
  4. US Department of Health and Human Services.^43,62,75^
  5. National Board of Health and Welfare.^76^
  6. Code of Federal Regulations and Health Insurance Portability and Accountability Act.^65^
2. Disclosure and Informed Consent Procedures
  Disclosure of relevant study and vaccine information:
  1. Information on vaccine provided in multiple languages.^27^
  2. Contacted by phone and provided study information.^74^
  3. Receipt of Vaccine Information Sheet (VIS) and/or study information sheet.^14,16,54,61,74^
  4. Posters and leaflets displayed at clinic/vaccination site.^61^
  5. Participants directed to bespoke study website.^61^
  6. Multistage community outreach campaign to disseminate information.^14^
  7. Telephone enquiry line available to answer questions.^37,61^
  8. Participants encouraged to speak with physician prior to enrollment.^19^
  9. Pharmacological counseling.^77^
  10. Verbal explanation.^78^
  Discussion and understanding of risk:
  1. Counseled on nature of project and risks involved.^53^
  2. Advised of the type, action, contraindications, adverse effects, and precautions.^20^
  3. Need for further health education to eliminate perceived safety concerns.^20^
  4. Participant understanding the importance of vaccination and risks of non-vaccination.^16^
  5. Advised to practice effective birth control and avoid pregnancy for specified period of time.^49^
  6. Need for requisite expertise to discuss ‘real time’ questions related to adverse events and different clinical scenarios.^29^
  Individual Informed Consent:
  1. Written consent obtained from study participant.^16,20,22,27,36-38,40,42,43,49,50,53-56,58,59,61-64,74,75,78,79^
  2. Written consent obtained from parent or guardian.^14,43^
  3. Consent given through online form.^61^
  4. Verbal consent.^14^
  5. Verbal assent.^14^
  6. Oral consent deemed appropriate-minimal risk to participant.^14^
  7. Study exempt from obtaining informed consent/assent.^39,48,52,60,65-71^
  Disclosure and informed consent of non-participants:
  1. Discussion with spouses and significant family members prior to obtaining informed consent.^50^
  2. Informed consent obtained from father of unborn child.^64,79^
  3. Community informed consent from local leaders.^14^
  Documentation of informed consent.^14^
3. Decision-making factors
  Safety Concerns:
  1. Participant fears of harmful effects on fetus and/or themselves.^16-28^
  2. Physician reluctance to vaccinate due to theoretical risks for fetus.^2,26,27^
  Perception of risk vs. benefit:
  1. Understanding of the importance of vaccination and risks of non-vaccination.^16^
  Outside influences:
  1. Role of friends and relatives in decision-making processes.^20^
  2. Societal pressures.^20^
  Prohibitive cost.^20^
  Product origins.^20^
4. Cultural considerations
  Providing information in culturally appropriate manner:
  1. Information provided in multiple languages.^27^
  Role of local leaders, community members, and relatives:
  1. Role of friends and relatives in decision-making processes.^20^
  2. Community informed consent from local leaders.^14^
  Local Customs:
  1. Inability to verbally confirm pregnancy during enrollment.^30^
5. Inadvertent immunization during pregnancy
  Unaware of pregnancy at time of vaccination.^51^
  Informed of risks post vaccine-exposure.^51^
  Awareness of risk:
  1. Aware that vaccination was contraindicated during pregnancy.^51^
  2. Unaware of potential risks of vaccination during pregnancy.^53^
  3. Counseling on periconceptional risks frequently omitted.^53^
6. Study design and enrollment
  Enrollment of pregnant women:
  1. Logistic and ethical challenges of excluding potentially pregnant women from mass vaccination campaigns.^14^
  2. Investigator reluctance to enroll pregnant women for fear of medical liability and theoretical safety concerns.^29^
  Ethical restrictions:
  1. Pregnant women considered a vulnerable population.^17,65^
  2. RCTs nearly impossible due to ethical considerations/guidelines.^25,77^
  Need for randomized controlled trials:
  1. Assess risk, safety, and adjust for bias.^2,18^
  2. Existing data from observational studies often does not reach standard for studies that are considered in determination of FDA pregnancy categories.^17^

Participant compensation.^19,54^

## References

[1] WHO UNICEF World Bank State of the world's vaccines and immunization. 3rd ed. 2009, Geneva: World Health Organization

[2] Makris MC, Polyzos KA, Mavros MN, Athanasiou S, Rafailidis PI, Falagas ME. Safety of hepatitis B, pneumococcal polysaccharide and meningococcal polysaccharide vaccines in pregnancy: a systematic review. Drug Saf 2012; 35(1):1-14; PMID:22149417; http://dx.doi.org/10.2165/11595670-000000000-00000 22149417

[3] Omer SB, Goodman D, Steinhoff MC, Rochat R, Klugman KP, Stoll BJ, Ramakrishnan U. Maternal influenza immunization and reduced likelihood of prematurity and small for gestational age births: a retrospective cohort study. PLoS Med 2011; 8(5):e1000441; PMID:21655318; http://dx.doi.org/10.1371/journal.pmed.1000441 21655318 PMC3104979

[4] Macroepidemiology of Influenza Vaccination Study, G. The macro-epidemiology of influenza vaccination in 56 countries, 1997–2003. Vaccine 2005; 23(44):5133-43; PMID:16039762; http://dx.doi.org/10.1016/j.vaccine.2005.06.010 16039762

[5] van Essen GA, Palache AM, Forleo E, Fedson DS. Influenza vaccination in 2000: recommendations and vaccine use in 50 developed and rapidly developing countries. Vaccine 2003; 21(16):1780-5; PMID:12686094; http://dx.doi.org/10.1016/S0264-410X(03)00072-0 12686094

[6] World Health Organization Vaccines against influenza WHO position paper – November 2012. Weekly Epidemiological Record 2012; 47(87):461-76

[7] Keller-Stanislawski B, Englund JA, Kang G, Mangtani P, Neuzil K, Nohynek H, Pless R, Lambach P, Zuber P. Safety of immunization during pregnancy: a review of the evidence of selected inactivated and live attenuated vaccines. Vaccine 2014; 32(52):7057-64; PMID:25285883; http://dx.doi.org/10.1016/j.vaccine.2014.09.052 25285883

[8] Lambach P, Hombach J, Ortiz JR. A global perspective of maternal immunization. Vaccine 2015; 33(47):6376-9; PMID:26319068; http://dx.doi.org/10.1016/j.vaccine.2015.08.036 26319068 PMC8243657

[9] US. Code of Federal Regulations 45 CFR 46 Protection of human subjects. 2009 11686173

[10] Foulkes MA, Grady C, Spong CY, Bates A, Clayton JA. Clinical research enrolling pregnant women: a workshop summary. J Womens Health (Larchmt) 2011; 20(10):1429-32; PMID:21819233; http://dx.doi.org/10.1089/jwh.2011.3118 21819233 PMC3186443

[11] Bhan A Influenza Vaccination in Pregnancy: Overview of ethical issues. 6 2011, Maternal Influenza Immunization Convening

[12] Lambach P, Alvarez AM, Hirve S, Ortiz JR, Hombach J, Verweij M, Hendriks J, Palkonyay L, Pfleiderer M. Considerations of strategies to provide influenza vaccine year round. Vaccine 2015; 33(47):6493-8; PMID:26319745; http://dx.doi.org/10.1016/j.vaccine.2015.08.037 26319745 PMC8218336

[13] Meeting of the Strategic Advisory Group of Experts on immunization, April 2015: conclusions and recommendations. Wkly Epidemiol Rec 2015; 90(22):261-78; PMID:26027016 26027016

[14] Hashim R, Khatib AM, Enwere G, Park JK, Reyburn R, Ali M, Chang NY, Kim DR, Ley B, Thriemer K, et al. Safety of the recombinant cholera toxin B subunit, killed whole-cell (rBS-WC) oral cholera vaccine in pregnancy. PLoS Negl Trop Dis 2012; 6(7):e1743; PMID:22848772; http://dx.doi.org/10.1371/journal.pntd.0001743 22848772 PMC3404114

[15] Fischer GW, Ottolini MG, Mond JJ. Prospects for vaccines during pregnancy and in the new born period. Clin Perinatol 1997; 24(1):231-49; PMID:9099512 9099512

[16] Auffret M, Béné J, Gautier S, Moreau-Crépeaux S, Caron J. Pharmacovigilance monitoring of a cohort of pregnant women vaccinated against influenza A(H1N1) variant virus in the Nord-Pas de Calais region of northern France. Eur J Obstet Gynecol Reprod Biol 2013; 170(1): p. 114-8; PMID:23810001; http://dx.doi.org/10.1016/j.ejogrb.2013.05.025 23810001

[17] Bednarczyk RA, Adjaye-Gbewonyo D, Omer SB. Safety of influenza immunization during pregnancy for the fetus and the neonate. Am J Obstet Gynecol 2012; 207(3 Suppl):S38-46; PMID:22920058; http://dx.doi.org/10.1016/j.ajog.2012.07.002 22920058

[18] Cantu J, Biggio J, Jauk V, Wetta L, Andrews W, Tita A. Selective uptake of influenza vaccine and pregnancy outcomes. J Matern Fetal Neonatal Med 2013; 26(12):1207-11; PMID:23406444; http://dx.doi.org/10.3109/14767058.2013.775419 23406444

[19] Christian LM, Iams JD, Porter K, Glaser R. Inflammatory responses to trivalent influenza virus vaccine among pregnant women. Vaccine 2011; 29(48):8982-7; PMID:21945263; http://dx.doi.org/10.1016/j.vaccine.2011.09.039 21945263 PMC3204610

[20] Huang G, Liu H, Cao Q, Liu B, Pan H, Fu C. Safety of post-exposure rabies prophylaxis during pregnancy: a follow-up study from Guangzhou, China. Hum Vaccin Immunother 2013; 9(1):177-83; PMID:23442589; http://dx.doi.org/10.4161/hv.22377 23442589 PMC3667934

[21] Kharbanda EO, Vazquez-Benitez G, Lipkind H, Naleway A, Lee G, Nordin JD. Inactivated influenza vaccine during pregnancy and risks for adverse obstetric events. Obstet Gynecol 2013; 122(3):659-67; PMID:23921876; http://dx.doi.org/10.1097/AOG.0b013e3182a1118a 23921876 PMC13445607

[22] Lin SY, Wu ET, Lin CH, Shyu MK, Lee CN. The Safety and Immunogenicity of Trivalent Inactivated Influenza Vaccination: A Study of Maternal-Cord Blood Pairs in Taiwan. PLoS One 2013; 8(6):e62983; PMID:23762229 23762229 10.1371/journal.pone.0062983 PMC3675132

[23] Moro PL, Tepper NK, Grohskopf LA, Vellozzi C, Broder K. Safety of seasonal influenza and influenza A (H1N1) 2009 monovalent vaccines in pregnancy. Expert Rev Vaccines 2012; 11(8):911-21; PMID:23002972 http://dx.doi.org/10.1586/erv.12.72 23002972

[24] Munoz FM, Greisinger AJ, Wehmanen OA, Mouzoon ME, Hoyle JC, Smith FA, Glezen WP. Safety of influenza vaccination during pregnancy. Am J Obstet Gynecol 2005; 192(4):1098-106; PMID:15846187; http://dx.doi.org/10.1016/j.ajog.2004.12.019 15846187

[25] Naleway AL, Irving SA, Henninger ML, Li DK, Shifflett P, Ball S, Williams JL, Cragan J, Gee J, Thompson MG, et al. Safety of influenza vaccination during pregnancy: A review of subsequent maternal obstetric events and findings from two recent cohort studies. Vaccine 2014; 32(26):3122-7; PMID:24742490; http://dx.doi.org/10.1016/j.vaccine.2014.04.021 24742490 PMC5898611

[26] Sheffield JS, Greer LG, Rogers VL, Roberts SW, Lytle H, McIntire DD, Wendel GD Jr. Effect of influenza vaccination in the first trimester of pregnancy. Obstet Gynecol 2012; 120(3):532-7; PMID:22914461; http://dx.doi.org/10.1097/AOG.0b013e318263a278 22914461

[27] Sheffield JS, Hickman A, Tang J, Moss K, Kourosh A, Crawford NM, Wendel GD Jr. Efficacy of an accelerated hepatitis B vaccination program during pregnancy. Obstet Gynecol 2011; 117(5):1130-5; PMID:21508752; http://dx.doi.org/10.1097/AOG.0b013e3182148efe 21508752

[28] Adedinsewo DA, Noory L, Bednarczyk RA, Steinhoff MC, Davis R, Ogbuanu C, Omer SB. Impact of maternal characteristics on the effect of maternal influenza vaccination on fetal outcomes. Vaccine 2013; 31(49):5827-33; PMID:24120677; http://dx.doi.org/; http://dx.doi.org/10.1016/j.vaccine.2013.09.071 24120677

[29] Sheffield JS, Munoz FM, Beigi RH, Rasmussen SA, Edwards KM, Read JS, Heine RP, Ault KA, Swamy GK, Jevaji I, et al. Research on vaccines during pregnancy: reference values for vital signs and laboratory assessments. Vaccine 2013; 31(40):4264-73; PMID:23906887; http://dx.doi.org/10.1016/j.vaccine.2013.07.031 23906887

[30] Lehmann D, Pomat WS, Riley ID, Alpers MP. Studies of maternal immunisation with pneumococcal polysaccharide vaccine in Papua New Guinea. Vaccine 2003; 21(24):3446-50; PMID:12850357; http://dx.doi.org/10.1016/S0264-410X(03)00348-7 12850357

[31] Ortiz JR, Neuzil KM, Ahonkhai VI, Gellin BG, Salisbury DM, Read JS, Adegbola RA, Abramson JS. Translating vaccine policy into action: a report from the Bill & Melinda Gates Foundation Consultation on the prevention of maternal and early infant influenza in resource-limited settings. Vaccine 2012; 30(50):7134-40; PMID:23026690; http://dx.doi.org/10.1016/j.vaccine.2012.09.034 23026690

[32] Fulton TR, Narayanan D, Bonhoeffer J, Ortiz JR, Lambach P, Omer SB. A systematic review of adverse events following immunization during pregnancy and the newborn period. Vaccine 2015; 33(47):6453-65; PMID:26413879; http://dx.doi.org/10.1016/j.vaccine.2015.08.043 26413879 PMC8290429

[33] Levac D, Colquhoun H, O'Brien KK. Scoping studies: advancing the methodology. Implement Sci 2010; 5:69; PMID:20854677; http://dx.doi.org/10.1186/1748-5908-5-69 20854677 PMC2954944

[34] Bonhoeffer J, Zumbrunn B, Heininger U. Reporting of vaccine safety data in publications: systematic review. Pharmacoepidemiol Drug Saf 2005; 14(2):101-6; PMID:15386705; http://dx.doi.org/10.1002/pds.979 15386705

[35] Strech D, Mertz M, Knüppel H, Neitzke G, Schmidhuber M. The full spectrum of ethical issues in dementia care: systematic qualitative review. Br J Psychiatry 2013; 202:400-6; PMID:23732935; http://dx.doi.org/10.1192/bjp.bp.112.116335 23732935

[36] Baker CJ, Rench MA, Edwards MS, Carpenter RJ, Hays BM, Kasper DL. Immunization of pregnant women with a polysaccharide vaccine of group B streptococcus. N Engl J Med 1988; 319(18):1180-5; PMID:3050524; http://dx.doi.org/10.1056/NEJM198811033191802 3050524

[37] Cavalcanti DP, Salomão MA, Lopez-Camelo J, Pessoto MA. Early exposure to yellow fever vaccine during pregnancy. Trop Med Int Health 2007; 12(7):833-7; PMID:17596249; http://dx.doi.org/10.1111/j.1365-3156.2007.01851.x 17596249

[38] Chambers CD, Johnson D, Xu R, Luo Y, Louik C, Mitchell AA, Schatz M, Jones KL. Risks and safety of pandemic H1N1 influenza vaccine in pregnancy: birth defects, spontaneous abortion, preterm delivery, and small for gestational age infants. Vaccine 2013; 31(44):5026-32; PMID:24016809; http://dx.doi.org/10.1016/j.vaccine.2013.08.097 24016809

[39] Conlin AM, Bukowinski AT, Sevick CJ, DeScisciolo C, Crum-Cianflone NF. Safety of the pandemic H1N1 influenza vaccine among pregnant US military women and their newborns. Obstet Gynecol 2013; 121(3):511-8; PMID:23635612; http://dx.doi.org/10.1097/AOG.0b013e318280d64e 23635612

[40] Jackson LA, Patel SM, Swamy GK, Frey SE, Creech CB, Munoz FM, Artal R, Keitel WA, Noah DL, Petrie CR, et al. Immunogenicity of an inactivated monovalent 2009 H1N1 influenza vaccine in pregnant women. J Infect Dis 2011; 204(6):854-63; PMID:21849282; http://dx.doi.org/10.1093/infdis/jir440 21849282 PMC3156926

[41] Lin TH, Lin SY, Lin CH, Lin RI, Lin HC, Chiu TH, Cheng PJ, Lee CN. AdimFlu-S((R)) influenza A (H1N1) vaccine during pregnancy: the Taiwanese Pharmacovigilance Survey. Vaccine 2012; 30(16):2671-5; PMID:22342546; http://dx.doi.org/10.1016/j.vaccine.2012.02.008 22342546

[42] Munoz FM, Bond NH, Maccato M, Pinell P, Hammill HA, Swamy GK, Walter EB, Jackson LA, Englund JA, Edwards MS, et al. Safety and immunogenicity of tetanus diphtheria and acellular pertussis (Tdap) immunization during pregnancy in mothers and infants: a randomized clinical trial. JAMA 2014; 311(17):1760-9; PMID:24794369; http://dx.doi.org/10.1001/jama.2014.3633 24794369 PMC4333147

[43] Munoz FM, Englund JA, Cheesman CC, Maccato ML, Pinell PM, Nahm MH, Mason EO, Kozinetz CA, Thompson RA, Glezen WP. Maternal immunization with pneumococcal polysaccharide vaccine in the third trimester of gestation. Vaccine 2001; 20(5-6):826-37; PMID:11738746; http://dx.doi.org/10.1016/S0264-410X(01)00397-8 11738746

[44] Munoz FM, Piedra PA, Glezen WP. Safety and immunogenicity of respiratory syncytial virus purified fusion protein-2 vaccine in pregnant women. Vaccine 2003; 21(24):3465-7; PMID:12850361; http://dx.doi.org/10.1016/S0264-410X(03)00352-9 12850361

[45] Nordin JD, Kharbanda EO, Benitez GV, Nichol K, Lipkind H, Naleway A, Lee GM, Hambidge S, Shi W, Olsen A. Maternal safety of trivalent inactivated influenza vaccine in pregnant women. Obstet Gynecol 2013; 121(3):519-25; PMID:23635613; http://dx.doi.org/10.1097/AOG.0b013e3182831b83 23635613

[46] Nordin JD, Kharbanda EO, Vazquez Benitez G, Lipkind H, Vellozzi C, Destefano F. Maternal influenza vaccine and risks for preterm or small for gestational age birth. J Pediatr 2014; 164(5):1051-1057 e2; PMID:24582484; http://dx.doi.org/10.1016/j.jpeds.2014.01.037 24582484

[47] Oppermann M, Fritzsche J, Weber-Schoendorfer C, Keller-Stanislawski B, Allignol A, Meister R, Schaefer C. A(H1N1)v2009: a controlled observational prospective cohort study on vaccine safety in pregnancy. Vaccine 2012; 30(30):4445-52; PMID:22564554; http://dx.doi.org/10.1016/j.vaccine.2012.04.081 22564554

[48] Pasternak B, Svanström H, Mølgaard-Nielsen D, Krause TG, Emborg HD, Melbye M, Hviid A. Risk of adverse fetal outcomes following administration of a pandemic influenza A(H1N1) vaccine during pregnancy. JAMA 2012; 308(2):165-74; PMID:22782418; http://dx.doi.org/10.1001/jama.2012.6131 22782418

[49] Pitisuttithum P, Rerks-Ngarm S, Bussaratid V, Dhitavat J, Maekanantawat W, Pungpak S, Suntharasamai P, Vanijanonta S, Nitayapan S, Kaewkungwal J, et al. Safety and reactogenicity of canarypox ALVAC-HIV (vCP1521) and HIV-1 gp120 AIDSVAX B/E vaccination in an efficacy trial in Thailand. PLoS One 2011; 6(12):e27837; PMID:22205930; http://dx.doi.org/10.1371/journal.pone.0027837 22205930 PMC3244387

[50] Quiambao BP, Nohynek HM, Käyhty H, Ollgren JP, Gozum LS, Gepanayao CP, Soriano VC, Makela PH. Immunogenicity and reactogenicity of 23-valent pneumococcal polysaccharide vaccine among pregnant Filipino women and placental transfer of antibodies. Vaccine 2007; 25(22):4470-7; PMID:17442467; http://dx.doi.org/10.1016/j.vaccine.2007.03.021 17442467

[51] Sato HK, Sanajotta AT, Moraes JC, Andrade JQ, Duarte G, Cervi MC, Curti SP, Pannuti CS, Milanez H, Pessoto M, et al. Rubella vaccination of unknowingly pregnant women: the Sao Paulo experience, 2001. J Infect Dis 2011; 204 Suppl 2:S737-44; PMID:21954275; http://dx.doi.org/10.1093/infdis/jir419 21954275

[52] Shakib JH, Korgenski K, Sheng X, Varner MW, Pavia AT, Byington CL. Tetanus, diphtheria, acellular pertussis vaccine during pregnancy: pregnancy and infant health outcomes. J Pediatr 2013; 163(5):1422-6 e1-4; PMID:23896191; http://dx.doi.org/10.1016/j.jpeds.2013.06.021 23896191 PMC4102585

[53] Suzano CE, Amaral E, Sato HK, Papaiordanou PM. The effects of yellow fever immunization (17DD) inadvertently used in early pregnancy during a mass campaign in Brazil. Vaccine 2006; 24(9):1421-6; PMID:16236402; http://dx.doi.org/10.1016/j.vaccine.2005.09.033 16236402

[54] Talbot EA, Brown KH, Kirkland KB, Baughman AL, Halperin SA, Broder KR. The safety of immunizing with tetanus-diphtheria-acellular pertussis vaccine (Tdap) less than 2 years following previous tetanus vaccination: Experience during a mass vaccination campaign of healthcare personnel during a respiratory illness outbreak. Vaccine 2010; 28(50):8001-7; PMID:20875487; http://dx.doi.org/10.1016/j.vaccine.2010.09.034 20875487

[55] Tavares F, Cheuvart B, Heineman T, Arellano F, Dubin G. Meta-analysis of pregnancy outcomes in pooled randomized trials on a prophylactic adjuvanted glycoprotein D subunit herpes simplex virus vaccine. Vaccine 2013; 31(13):1759-64; PMID:23313657; http://dx.doi.org/10.1016/j.vaccine.2013.01.002 23313657

[56] Zaman K, Roy E, Arifeen SE, Rahman M, Raqib R, Wilson E, Omer SB, Shahid NS, Breiman RF, Steinhoff MC. Effectiveness of maternal influenza immunization in mothers and infants. N Engl J Med 2008; 359(15):1555-64; PMID:18799552; http://dx.doi.org/10.1056/NEJMoa0708630 18799552

[57] De Vries L, et al. Adjuvanted A/H1N1 (2009) influenza vaccination during pregnancy: Description of a prospective cohort and spontaneously reported pregnancy-related adverse reactions in the Netherlands. Birth Defects Research Part A - Clinical and Molecular Teratology 2014 ((De Vries L., l.devries@lareb.nl; van Hunsel F; Cuppers-Maarschalkerweerd B; van Puijenbroek E.) Netherlands Pharmacovigilance Centre Lareb's-Hertogenbosch the Netherlands) 10.1002/bdra.23243 24706475

[58] Heikkinen T, Young J, van Beek E, Franke H, Verstraeten T, Weil JG, Della Cioppa G. Safety of MF59-adjuvanted A/H1N1 influenza vaccine in pregnancy: a comparative cohort study. Am J Obstet Gynecol 2012; 207(3):177 e1-8; PMID:22939717; http://dx.doi.org/10.1016/j.ajog.2012.07.007 22939717

[59] Horiya M, Hisano M, Iwasaki Y, Hanaoka M, Watanabe N, Ito Y, Kojima J, Sago H, Murashima A, Kato T, Yamaguchi K. Efficacy of double vaccination with the 2009 pandemic influenza A (H1N1) vaccine during pregnancy. Obstet Gynecol 2011; 118(4):887-94; PMID:21934453; http://dx.doi.org/10.1097/AOG.0b013e31822e5c02 21934453

[60] Ludvigsson JF, Zugna D, Cnattingius S, Richiardi L, Ekbom A, Örtqvist Å, Persson I, Stephansson O. Influenza H1N1 vaccination and adverse pregnancy outcome. Eur J Epidemiol 2013; 28(7):579-88; PMID:23715672; http://dx.doi.org/10.1007/s10654-013-9813-z 23715672

[61] Mackenzie IS, MacDonald TM, Shakir S, Dryburgh M, Mantay BJ, McDonnell P, Layton D. Influenza H1N1 (swine flu) vaccination: a safety surveillance feasibility study using self-reporting of serious adverse events and pregnancy outcomes. Br J Clin Pharmacol 2012; 73(5):801-11; PMID:22082196; http://dx.doi.org/10.1111/j.1365-2125.2011.04142.x 22082196 PMC3403208

[62] Santosham M, Englund JA, McInnes P, Croll J, Thompson CM, Croll L, Glezen WP, Siber GR. Safety and antibody persistence following Haemophilus influenzae type b conjugate or pneumococcal polysaccharide vaccines given before pregnancy in women of childbearing age and their infants. Pediatr Infect Dis J 2001; 20(10):931-40; PMID:11642626; http://dx.doi.org/10.1097/00006454-200110000-00005 11642626

[63] Tavares F, Nazareth I, Monegal JS, Kolte I, Verstraeten T, Bauchau V. Pregnancy and safety outcomes in women vaccinated with an AS03-adjuvanted split virion H1N1 (2009) pandemic influenza vaccine during pregnancy: a prospective cohort study. Vaccine 2011; 29(37):6358-65; PMID:21596080; http://dx.doi.org/10.1016/j.vaccine.2011.04.114 21596080

[64] Wright P, Lambert JS, Gorse GJ, Hsieh RH, McElrath MJ, Weinhold K, Wara DW, Anderson EL, Keefer MC, Jackson S, et al. Immunization with envelope MN rgp120 vaccine in human immunodeficiency virus-infected pregnant women. J Infect Dis 1999; 180(4):1080-8; PMID:10479134; http://dx.doi.org/10.1086/314985 10479134

[65] Dana A, Buchanan KM, Goss MA, Seminack MM, Shields KE, Korn S, Cunningham ML, Haupt RM. Pregnancy outcomes from the pregnancy registry of a human papillomavirus type 6/111487;161487;18 vaccine. Obstet Gynecol 2009; 114(6):1170-8; PMID:19935016; http://dx.doi.org/10.1097/AOG.0b013e3181c2a122 19935016

[66] Moro PL, Broder K, Zheteyeva Y, Revzina N, Tepper N, Kissin D, Barash F, Arana J, Brantley MD, Ding H, et al. Adverse events following administration to pregnant women of influenza A (H1N1) 2009 monovalent vaccine reported to the Vaccine Adverse Event Reporting System. Am J Obstet Gynecol 2011; 205(5):473 e1-9; PMID:21861964; http://dx.doi.org/10.1016/j.ajog.2011.06.047 21861964 PMC6602056

[67] Moro PL, Broder K, Zheteyeva Y, Walton K, Rohan P, Sutherland A, Guh A, Haber P, Destefano F, Vellozzi C, et al. Adverse events in pregnant women following administration of trivalent inactivated influenza vaccine and live attenuated influenza vaccine in the Vaccine Adverse Event Reporting System, 1990-2009. Am J Obstet Gynecol 2011; 204(2):146 e1-7; PMID:20965490; http://dx.doi.org/10.1016/j.ajog.2010.08.050 20965490

[68] Moro PL, Museru OI, Broder K, Cragan J, Zheteyeva Y, Tepper N, Revzina N, Lewis P, Arana J, Barash F, et al. Safety of influenza A (H1N1) 2009 live attenuated monovalent vaccine in pregnant women. Obstet Gynecol 2013; 122(6):1271-8; PMID:24201689; http://dx.doi.org/10.1097/AOG.0000000000000010 24201689

[69] Moro PL, Museru OI, Niu M, Lewis P, Broder K. Reports to the Vaccine Adverse Event Reporting System after hepatitis A and hepatitis AB vaccines in pregnant women. Am J Obstet Gynecol 2014; 210(6):561 e1-6; PMID:24378675; http://dx.doi.org/10.1016/j.ajog.2013.12.036 24378675 PMC6500450

[70] Zheteyeva Y, Moro PL, Yue X, Broder K. Safety of meningococcal polysaccharide-protein conjugate vaccine in pregnancy: a review of the Vaccine Adverse Event Reporting System. Am J Obstet Gynecol 2013; 208(6):478 e1-6; PMID:23453881; http://dx.doi.org/10.1016/j.ajog.2013.02.027 23453881

[71] Zheteyeva YA, Moro PL, Tepper NK, Rasmussen SA, Barash FE, Revzina NV, Kissin D, Lewis PW, Yue X, Haber P, et al. Adverse event reports after tetanus toxoid, reduced diphtheria toxoid, and acellular pertussis vaccines in pregnant women. Am J Obstet Gynecol 2012; 207(1):59 e1-7; PMID:22727350; http://dx.doi.org/10.1016/j.ajog.2012.05.006 22727350

[72] Toback SL, Beigi R, Tennis P, Sifakis F, Calingaert B, Ambrose CS. Maternal outcomes among pregnant women receiving live attenuated influenza vaccine. Influenza Other Respir Viruses 2012; 6(1):44-51; PMID:21672166; http://dx.doi.org/10.1111/j.1750-2659.2011.00266.x 21672166 PMC4941557

[73] Orenstein LA, Orenstein EW, Teguete I, Kodio M, Tapia M, Sow SO, Levine MM. Background Rates of Adverse Pregnancy Outcomes for Assessing the Safety of Maternal Vaccine Trials in Sub-Saharan Africa. PLoS One 2012; 7(10):e46638; PMID:23056380; http://dx.doi.org/10.1371/journal.pone.0046638 23056380 PMC3464282

[74] Launay O, Krivine A, Charlier C, Truster V, Tsatsaris V, Lepercq J, Ville Y, Avenell C, Andrieu T, Rozenberg F, et al. Low rate of pandemic A/H1N1 2009 influenza infection and lack of severe complication of vaccination in pregnant women: a prospective cohort study. PLoS One 2012; 7(12):e52303; PMID:23300637; http://dx.doi.org/10.1371/journal.pone.0052303 23300637 PMC3531481

[75] Abzug MJ, Nachman SA, Muresan P, Handelsman E, Watts DH, Fenton T, Heckman B, Petzold E, Weinberg A, Levin MJ. Safety and immunogenicity of 2009 pH1N1 vaccination in HIV-infected pregnant women. Clin Infect Dis 2013; 56(10):1488-97; PMID:23378284; http://dx.doi.org/10.1093/cid/cit057 23378284 PMC3634309

[76] Kallen B, Olausson PO. Vaccination against H1N1 influenza with Pandemrix((R)) during pregnancy and delivery outcome: a Swedish register study. BJOG 2012; 119(13):1583-90; PMID:22901103; http://dx.doi.org/10.1111/j.1471-0528.2012.03470.x 22901103

[77] Ergenoglu AM, Yeniel AO, Yildirim N, Kazandi M, Akercan F, Sağol S. Rubella vaccination during the preconception period or in pregnancy and perinatal and fetal outcomes. Turk J Pediatr 2012; 54(3):230-3; PMID:23094531 23094531

[78] Chavant F, Ingrand I, Jonville-Bera AP, Plazanet C, Gras-Champel V, Lagarce L, Zenut M, Disson-Dautriche A, Logerot S, Auffret M, et al. The PREGVAXGRIP study: a cohort study to assess foetal and neonatal consequences of in utero exposure to vaccination against A(H1N1)v2009 influenza. Drug Saf 2013; 36(6):455-65; PMID:23516007; http://dx.doi.org/10.1007/s40264-013-0030-1 23516007

[79] Baker CJ, Rench MA, McInnes P. Immunization of pregnant women with group B streptococcal type III capsular polysaccharide-tetanus toxoid conjugate vaccine. Vaccine 2003; 21(24):3468-72; PMID:12850362; http://dx.doi.org/10.1016/S0264-410X(03)00353-0 12850362

