# Supplementary material for: A systematic review of ethical issues in vaccine studies involving pregnant women
Source: Hum Vaccin Immunother. 2016 May 31;12(8):1952–9. doi: 10.1080/21645515.2016.1186312 (PMC4994733; doi:10.1080/21645515.2016.1186312)
Supplement: Supplemental_Material.docx [file khvi-12-08-1186312-s001.docx]

**Supplementary Materials**

| **Supplementary Table 1 Characteristics of selected publications** | | | |
| --- | --- | --- | --- |
| **Study author and year [reference]** | **Continent** | **Study design** | **Type of vaccine** |
| **Abzug 2013 [75]** | North America | Prospective cohort | Influenza |
| **Adedinsewo 2013 [28]** | North America | Retrospective cohort | Influenza |
| **Auffret 2013 [16]** | Europe | Prospective cohort | Influenza |
| **Baker 1988 [36]** | North America | Prospective cohort | GBS |
| **Baker 2003 [79]** | North America | RCT | GBS |
| **Bednarczyk 2012 [17]** | Multiple | Review | Influenza |
| **Black 2004** | North America | Retrospective cohort | Influenza |
| **Cantu 2013 [18]** | North America | Retrospective cohort | Influenza |
| **Cavalcanti 2007 [37]** | South America | Before/after | Yellow fever |
| **Chambers 2013 [38]** | North America | Prospective cohort | Influenza |
| **Chavant 2013 [78]** | Europe | Prospective cohort | Influenza |
| **Christian 2011 [19]** | North America | Prospective cohort | Influenza |
| **Conlin 2013 [39]** | North America | Retrospective cohort | Influenza |
| **Cottin 2013** | Multiple | Review | Yellow fever |
| **da Silva 2011** | South America | Prospective cohort | Rubella |
| **Dana 2009 [65]** | Multiple | Prospective cohort | HPV |
| **De Vries 2014 [57]** | Europe | Prospective cohort | Influenza |
| **Ergenoglu 2012 [77]** | Europe | Prospective cohort | Rubella |
| **Harjulehto-Mervaala 1994** | Europe | Prospective cohort | OPV |
| **Hashim 2012 [14]** | Africa | Cross-sectional | Cholera |
| **Heikkinen 2012 [58]** | Multiple | Prospective cohort | Influenza |
| **Horiya 2011 [59]** | Asia | RCT | Influenza |
| **Huang 2013 [20]** | Asia | Prospective cohort | Rabies |
| **Jackson 2011 [40]** | North America | Prospective cohort | Influenza |
| **Kallen 2012 [76]** | Europe | Retrospective cohort | Influenza |
| **Kharbanda 2012 [21]** | North America | Prospective cohort | Influenza |
| **Kharbanda 2013** | North America | Retrospective cohort | Influenza |
| **Launay 2012 [74]** | Europe | Prospective cohort | Influenza |
| **Lehmann 2003 [30]** | Asia | Review | Pneumococcal |
| **Lin 2012 [22]** | Asia | Retrospective cohort | Influenza |
| **Lin 2013 [41]** | Asia | Prospective cohort | Influenza |
| **Ludvigsson 2013 [60]** | Europe | Retrospective cohort | Influenza |
| **Mackenzie 2012 [61]** | Europe | Prospective cohort | Influenza |
| **Makris 2012 [2]** | Multiple | Review | Multiple |
| **Moro 2011 [66]** | North America | Retrospective cohort | Influenza (monovalent) |
| **Moro 2011 [67]** | North America | Retrospective cohort | Influenza (trivalent) |
| **Moro 2012 [68]** | Multiple | Review | Influenza |
| **Moro 2013 [69]** | North America | Retrospective cohort | Influenza |
| **Moro 2014 [23]** | North America | Retrospective cohort | Hepatitis |
| **Munoz 2001 [42]** | North America | RCT | Multiple |
| **Munoz 2003 [43]** | North America | RCT | RSV |
| **Munoz 2005 [24]** | North America | Retrospective cohort | Influenza |
| **Munoz 2014 [44]** | North America | RCT | Tdap |
| **Naleway 2014 [25]** | North America | Review | Influenza |
| **Nishioka 1998** | South America | Case control | Yellow fever |
| **Nordin 2013 [45]** | North America | Retrospective cohort | Influenza |
| **Nordin 2014 [46]** | North America | Retrospective cohort | Influenza |
| **Omon 2011** | Europe | Prospective cohort | Influenza |
| **Oppermann 2012 [47]** | Europe | Prospective cohort | Influenza |
| **Orenstein 2012 [73]** | Africa | Review | Multiple |
| **Pardon 2011** | South America | Prospective cohort | Rubella |
| **Pass 2009** | North America | RCT | CMV |
| **Pasternak 2012 [48]** | Europe | Retrospective cohort | Influenza |
| **Pasternak 2012 [48]** | Europe | Retrospective cohort | Influenza |
| **Pitisuttithum 2011 [49]** | Asia | RCT | HIV |
| **Quiambao 2007 [50]** | Asia | RCT | Pneumococcal |
| **Santosham 2001 [62]** | North America | RCT | Multiple |
| **Sato 2011 [51]** | South America | Prospective cohort | Rubella |
| **Shakib 2013 [52]** | North America | Retrospective cohort | Tdap |
| **Sheffield 2011 [26]** | North America | Prospective cohort | Hepatitis |
| **Sheffield 2012 [27]** | North America | Retrospective cohort | Influenza |
| **Sheffield 2013 [29]** | Multiple | Review | Multiple |
| **Silveira 1995** | South America | Case control | TT |
| **Suzano 2006 [53]** | South America | Retrospective cohort | Yellow fever |
| **Talbot 2010 [54]** | North America | Cross-sectional | Tdap |
| **Tavares 2011 [55]** | Europe | Prospective cohort | Influenza |
| **Tavares 2013 [63]** | Multiple | Review | Herpes simplex |
| **Thomas 2012** | Multiple | Review | Yellow fever |
| **Toback 2012 [72]** | North America | Retrospective cohort | Influenza |
| **Tsai 2010** | Europe | Retrospective cohort | Influenza |
| **Wilson 2008** | North America | Retrospective cohort | Varicella |
| **Wise 2000** | North America | Retrospective cohort | Varicella |
| **Wright 1999 [64]** | North America | RCT | HIV |
| **Zaman 2008 [56]** | Asia | RCT | Influenza |
| **Zheteyeva 2012 [70]** | North America | Retrospective cohort | Tdap |
| **Zheteyeva 2013 [71]** | North America | Retrospective cohort | Meningococcal |

**Additional References:**

Black, S.B., et al., *Effectiveness of influenza vaccine during pregnancy in preventing hospitalizations and outpatient visits for respiratory illness in pregnant women and their infants.* Am J Perinatol, 2004. 21(6): p. 333-9.

Cottin, P., M. Niedrig, and C. Domingo, *Safety profile of the yellow fever vaccine Stamaril(registered trademark): A 17-year review.* Expert Rev Vaccines, 2013. 12(11): p. 1351-1368.

da Silva e Sa, G.R., et al., *Pregnancy outcomes following rubella vaccination: a prospective study in the state of Rio de Janeiro, Brazil, 2001-2002.* J Infect Dis, 2011. 204 Suppl 2: p. S722-8.

Harjulehto-Mervaala, T., et al., *Oral polio vaccination during pregnancy: lack of impact on fetal development and perinatal outcome.* Clin Infect Dis, 1994. 18(3): p. 414-20.

Kharbanda, E.O., et al., *Assessing the safety of influenza immunization during pregnancy: the Vaccine Safety Datalink.* Am J Obstet Gynecol, 2012. 207(3 Suppl): p. S47-51.

Nishioka Sde, A., et al., *Yellow fever vaccination during pregnancy and spontaneous abortion: a case-control study.* Trop Med Int Health, 1998. 3(1): p. 29-33.

Omon, E., et al., *Non-adjuvanted 2009 influenza A (H1N1)v vaccine in pregnant women: the results of a French prospective descriptive study.* Vaccine, 2011. 29(52): p. 9649-54.

Pardon, F., et al., *Rubella vaccination of unknowingly pregnant women during 2006 mass campaign in Argentina.* J Infect Dis, 2011. 204 Suppl 2: p. S745-7.

Pass, R.F., et al., *Vaccine prevention of maternal cytomegalovirus infection.* N Engl J Med, 2009. 360(12): p. 1191-9.

Silveira, C.M., et al., *Safety of tetanus toxoid in pregnant women: a hospital-based case-control study of congenital anomalies.* Bull World Health Organ, 1995. 73(5): p. 605-8.

Thomas, R.E., et al., *The safety of yellow fever vaccine 17D or 17DD in children, pregnant women, HIV+ individuals, and older persons: systematic review.* Am J Trop Med Hyg, 2012. 86(2): p. 359-72.

Tsai, T., et al., *Exposure to MF59-adjuvanted influenza vaccines during pregnancy--a retrospective analysis.* Vaccine, 2010. 28(7): p. 1877-80.

Wilson, E., et al., *Varicella vaccine exposure during pregnancy: data from 10 Years of the pregnancy registry.* J Infect Dis, 2008. 197 Suppl 2: p. S178-84.

Wise, R.P., et al., *Postlicensure safety surveillance for varicella vaccine.* JAMA, 2000. 284(10): p. 1271-9.
